# Association Between Self-Perceived Stigma and Quality of Life Among Urban Chinese Older Adults: The Moderating Role of Attitude Toward Own Aging and Traditionality

**DOI:** 10.3389/fpubh.2022.767255

**Published:** 2022-02-11

**Authors:** Tao Sun, Shu-E Zhang, Meng-yao Yan, Ting-hui Lian, Yi-qi Yu, Hong-yan Yin, Chen-xi Zhao, Yan-ping Wang, Xiao Chang, Ke-yu Ji, Si-yu Cheng, Xiao-he Wang, Xian-hong Huang, De-pin Cao

**Affiliations:** ^1^Department of Health Policy and Management, School of Public Health, Hang Zhou Normal University, Hangzhou, China; ^2^Department of Health Management, School of Health Management, Harbin Medical University, Harbin, China; ^3^Department of Humanities and Social Sciences, Harbin Medical University, Daqing, China

**Keywords:** self-perceived stigma, attitude toward own aging, traditionality, quality of life, aging

## Abstract

**Background:**

Ageism is a global challenge, which leads to a range of adverse outcomes for elderly people worldwide, which maybe more severe among urban older adults in a competitive society. However, how self-perceived ageism influences the quality of life in a sample of urban older adults remains inconclusive.

**Objectives:**

The current study aims to assess the status of self-perceived stigma among urban Chinese older adults, identify its relationship with quality of life, and further explore whether both attitude toward own aging and traditionality moderate this relationship.

**Materials and Methods:**

Primary data were collected through cross-sectional surveys among urban older adults in three provinces of China from October 2019 to December 2020. A total of 764 urban older adults were valid participants (effective response rate = 81.28%) and completed questionnaires via anonymous face-to-face interviews. Socio-demographic factors, self-perceived stigma, attitude toward own aging, traditionality, and quality of life were assessed using questionnaires that included the Self-perceived Stigma, Attitude Toward Own Aging, Traditionality, and SF-8 Scales.

**Results:**

For urban Chinese older adults, the average score of self-perceived stigma was 2.041 ± 0.726. Self-perceived stigma (β = −0.391, *p* < 0.05) and attitude toward own aging (β = −0.211, *p* < 0.05) both influenced quality of life. Additionally, attitude toward own aging (β = −0.530, *p* < 0.05) and traditionality (β = −0.525, *p* < 0.05) moderated the association between self-perceived stigma and quality of life. Simple slope analysis revealed that when the level of negative attitude toward own aging and traditionality was higher, the strength of the influence of self-perceived stigma on quality of life was stronger.

**Conclusion:**

Urban Chinese older adults were aware of the self-perceived stigma, which contributes to decreased quality of life. Attitude toward own aging and traditionality could moderate the association between self-perceived stigma and quality of life. When negative attitudes toward own aging and traditionality are higher, self-perceived stigma has a greater effect on the quality of life. More interventions related to relieving self-perceived stigma, traditionality, and negative attitude toward own aging should be considered to build a new modern society that emphasizes health, friendliness, well-being, and dignity for all ages.

## Introduction

In many modern “youth-centered” societies worldwide, discrimination against older people, regarded as ageism, is common. Ageism refers to stereotypes, prejudice, and discrimination against a particular age group, especially the elderly (1). A recent report showed that experiencing ageism is prone to lead to poorer health, social isolation, earlier deaths, and economic consequences worth billions; therefore, swift action implementing effective anti-ageism strategies is required (2). Older adults unavoidably encounter a period of biological and physical deterioration, including changes in body posture, hair color, voice, and ability to see and hear (3); these contribute to public stereotypes, prejudice, and discrimination toward older adults in daily life (4, 5). Especially in the digital age, there is a growing gap between older and younger individuals regarding values, thoughts, and lifestyles (6). Further, whereas social forces driven by the transformation of new media, social networking technology, and marketing strategies contribute to encouraging youth-centered lifestyle, they shape negative attitudes toward aging and elicit adverse depictions regarding older adults and the aging process (7, 8). Numerous older adults also struggle with multiple dilemmas and are often stereotyped as forgetful, useless, lonely, and unattractive (9). Ageism can not only change how older adults view themselves but can also erode solidarity between generations and devalue or limit public ability to benefit from younger and older populations, further increasing the threat to everybody's health, longevity, and well-being (10); there are also far-reaching impacts on economies and societies.

Self-perceived stigma and attitude toward own aging as two different patterns together make up one set of the ageism (11). Self-perceived stigma presents an age-based stereotype threat, meaning that older adults perceive age-based stigma from other social groups. Differently, the attitude toward own aging of elderly presents older adults' internal views on aging, as self-imposed ageism, meaning that it consists of subjective beliefs of older adult individual toward own cognitive abilities and physical capabilities. The difference of two concepts has been discussed in the previous literature (12) Since the concept of self-perceived stigma was proposed, there have been studies on different groups of people, such as people with mental illnesses (13), people with HIV (14), Chinese female sex workers and Chinese breast cancer survivors (15), and obesity-related perceived stigma (16).

Existing literature indicated that stigma was widespread, and its associated consequences were far-reaching, including lower quality of life, earlier death, poorer physical and mental health, and slower recovery from disability in older age (8, 17). Due to existing deterioration in physical function, appearance, and competence; lower ability to contribute to society; and social exit and retirement, it is difficult for older adults to keep lasting work and live happily or achieve a long-lasting, adequate quality of life (9), which refers to an individual's perception or evaluation toward self-position in the space of everyday social life.

Quality of life is nested inside the specific cultural context and value system one depends on for survival and is related to a set of goals, expectations, standards, and concerns (18); it is a multidimensional concept that consists of both objective and subjective domains, macro and micro-aspects, and positive and negative components (19). Moreover, quality of life is a reliable and relevant indicator of older people's health and well-being (19), taking into account physical and psychological functions, body activity and participation, and environmental domains (20).

### Relationship of Self-Perceived Stigma and Quality of Life

Several similar studies demonstrated that stigma caused psychological distress and social withdrawal, leading to decreased quality of life in different groups of patients, such as those with depressive disorders (21), Parkinson's disease (22), and HIV (23). Moreover, a meta-analysis showed that self-concept and social networks play a mediating role in the association between stigma and quality of life in patients with psychosis (24). Undoubtedly, age-based stereotypes or external discrimination increase the emergence of internal stigma and self-directed ageism (25) that also seriously threaten the well-being of older people. However, few studies have been conducted on stigma among general older adults, especially in China. Moreover, the potential mechanism of the association between perceived stigma and quality of life remains inconclusive. Previous studies confirmed that age-based stereotypes, prejudice, and discrimination harmed older adults, thus severely limiting opportunities to secure their health, well-being, and quality of life (8).

### The Moderating Effect of Attitudes Toward Aging in the Relationship

A review study revealed that self-perceived stigma of older adults as an other-directed ageism has a negative impact on the physical and mental health (26). In fact, some older adults don't be affected although in the same social ecosystem in which stigmatization related to age (i.e., ageism) is a widespread phenomenon in the modern industrial societies (26). Now, we suspect that there is difference in individuals who present differentiated internal views on own aging. Several studies have established the associations between perceived stigma and aging attitudes and quality of life (17). Traditionally, understanding attitudes toward aging in various cultural contexts is crucial to foster the optimal quality of life for older adults (27). Existing studies have measured older adults' attitudes toward aging as stable and integrative judgments, summarizing their thoughts, feelings, and memories regarding aging or other situations; further, the subjective perception of aging, rather than the individual's chronological age and life, are emphasized (27). Generally, a significant association between elderly people's attitude toward own aging and the state of physical and mental health (28), as well as life satisfaction and well-being (29), has been found. Moreover, a previous study has revealed the negative impact of internalized stigma on the quality of life of people with serious mental (30) and chronic (31) illnesses. In addition, a recent study found that attitude toward own aging was a potential mediator in the relationship between personality factors and mental health and life satisfaction among older adults (32). Another study reported that attitude to aging moderated the relationship between subjective age and psychological well-being (33). A study regarding the COVID-19 pandemic showed that subjective age moderated the negative association between ageism and subjective health (10). In conclusion, attitude to aging likely moderates the association between self-perceptive stigma and quality of life among older adults. Integrating the evidence presented above regarding existing tendencies among older people, it can be surmised that attitude toward own aging can strengthen the correlation between self-perceived stigma and quality of life; this potentially contributes to providing a new clue for social governance to improve older adults' quality of life in the current aging society.

### The Moderating Effect of Traditionality in the Relationship

Previous study suggested that the association varies across cultures shaped by a series of traditions, religious and sociocultural beliefs, and modern lifestyles (34). Further, traditionality can always be found in modern Chinese society; the ubiquity of traditionality may be related to Confucian teaching, which emphasizes interdependence and group harmony. However, whether traditionality potentially influences the strength of the associations discussed above remains unknown. Considering most past studies excluded cultural factors, the current study aims to provide meaningful insight regarding the interaction between culture and stigmatization and how this influences the quality of life of Chinese older adults. In China, due to the existing highly collective culture, traditionality is regarded as “an emphasis on expressive ties among people and values, such as respect for authority, filial piety, ancestor worship, male-domination, fatalism, and a general sense of powerlessness (35).” Chinese adults used to follow the five basic relations formed by Confucianism in traditional Chinese culture: emperor-subject, father-son, husband-wife, elder-younger, and friend-friend (35). Older adults, influenced by the existence of historical and human-cultural factors, used to exhibit high degrees of traditionality compared to younger individuals.

Yang defined Chinese traditionality as “the typical pattern of more or less related motivational, evaluative, attitudinal and temperamental traits that is most frequently observed in people in traditional Chinese society and can still be found in people in contemporary Chinese societies such as Taiwan, Hong Kong, and mainland China” (36). Generally, the traditionality refers to the extent to which individuals adhere to traditional cultural beliefs and values (37), which will deeply determinate person's mindset pattern and behavioral style, as well lifestyle. As such, older adults are likely prone to emphasize the extent to which individuals should fulfill the expectations defined by prescribed social roles based on the five basic relations (38). Conversely, older adults with low-degree traditionality are more likely to exhibit egalitarianism, self-reliance, and openness. Under the impact of the high level of traditionality, self-stigmatizing older adults are more likely to carry out limited resistance against ageism, resulting in an incapable adjustment from negative to positive emotions (35). Older adults with a high degree of traditionality are more likely to contribute to the limited quality of everyday communication with other community members in modern society, further restraining social interaction and positive support from others, hinting that high-degree traditionality potentially leads to lower quality of life (38). An latest study in Italy revealed that the traditionalism was a determinant in the emergence of adolescents' ageism toward older adults (39). A cross-cultural meta-analysis suggested that the determinant of regional differences played an important role in shaping modern attitudes toward elders, further presenting that in modern, industrialized societies, easterners with collectivist traditions present negative attitude toward elders (40). Conversely, cultural individualism significantly predicted relative positivity-suggesting that, for generating elder respect within rapidly aging societies (40).

In general, the current study assumed that low degrees of traditionality can buffer the destructive effects of self-perceived stigma on quality of life. Moreover, existing research shows that traditionality plays a moderating role in a series of associations in the Chinese context (38, 41). Therefore, the current research attempted to explore the moderating effect of traditionality in the relationship between self-perceived stigma and quality of life among Chinese older adults.

### Aim

This study aimed to enhance the understanding of self-perceived stigma among urban Chinese older adults and to further explore the moderating mechanism of its relation to the quality of life. Specially, the current study aimed to examine the prevalence of self-perceived stigma and attitude toward own aging, and to probe the moderating role in the relationship between them, including attitude toward own aging and traditionality.

## Materials and Methods

### Sample and Data Collection

Primary data were collected using a cross-sectional survey from three provinces in China. A multistage stratified convenience sampling was used. Three provinces included northeast China' Heilongjiang province, east China's Zhejiang province and southeast China' Guangdong province, well-trained investigators randomly chooses communities and surveyed the encounter older adults at different times of the day. Participation to the current study was voluntary, informed consent was obtained from all participants on the front page of the self-administered questionnaires.

Considering the calculation method and standard requirements of the cross-sectional sample size from Zhou et al. (42), we identified 224 as the minimum sample size for this study. Of the 940 eligible patients, 176 were excluded. There were 764 urban older adults who were valid participants (effective response rate = 81.28%) and completed questionnaires via face-to-face interviews from October 2019 to December 2020. Face-to-face interviews were conducted by well-trained investigators among urban older adults. A brief interaction begins with an elucidation of research purpose or subject inclusion criteria, followed by inquiries or evaluation to address these criteria in increasingly precise ways on selecting participants. The eligibility criteria were being aged 60 years or older, having resided in the city for more than 6 months, being able to both listen and talk with others in Chinese, no diagnosis of Dementia, having clear cognition, having no serious hearing impairments, and having volunteered to participate in the research. In the current study, the basic demographic characteristics were consistent with the Chinese official data.

#### Measurement of Self-Perceived Stigma

The five-item Chinese version of the Self-perceived Stigma Scale was used to measure the self-perceived stigma of urban Chinese older adults (43); this scale has been proven to have good reliability and validity among Chinese subjects. Original questions have been subtly modified to suit this survey which assessed attitudes toward urban older adults in the Chinese context. Responses were graded on a five-point Likert scale (1 = not at all, 5 = very frequent). Sample items included “Because of my age, I felt emotionally distant from other people.” The total score ranged from 5 to 25, with higher scores reflecting higher self-perceived stigma of urban older adults. In the current study, Cronbach's alpha coefficient was 0.791.

#### Measurement of Attitude Toward Own Aging

Attitude toward own aging was measured using a five-item questionnaire derived from the short version of the Attitudes to Aging Questionnaire (44), which has been widely used in the Chinese context (45, 46). Responses were graded on a five-point Likert scale (1 = completely disagree, 5 = completely agree). Sample items included “I think I am old”; “In my opinion, getting old is a process of constant loss”; “As I get older, I find it more difficult to make new friends”; and “Due to my age, I feel excluded.” The total score ranged from 5 to 25, with higher scores reflecting higher negative attitude toward own aging among urban older adults. The scale had qualified internal consistency, with an overall Cronbach's alpha of 0.735 in this study.

#### Measurement of Traditionality

The Traditionality Scale was a five-item questionnaire originally developed by Yang et al. (35) and applied by Farh (41, 47), which has been widely used in the Chinese context (38, 48). Responses were evaluated using a five-point Likert scale (1 = strongly disagree, 5 = strongly agree). The total score ranged from 5 to 25, with higher scores reflecting higher traditionality. Sample items included “The chief government official is like the head of a household, the citizen should obey his decisions on all state matters”; “The best way to avoid mistakes is to follow the instructions of senior persons”; “When people are in dispute, they should ask the most senior person to decide who is right”; “Children should respect those people who are respected by their parents”; and “Before marriage, a woman should subordinate herself to her father, and after marriage, to her husband.” The scale had acceptable internal consistency, with Cronbach's alpha of 0.782 for this study.

#### Measurement of Health-Related Quality of Life

Considering the cost-effectiveness and accessibility of the tool, the eight-item version of the SF Health Survey (SF-8) was used to evaluate the quality of life among urban older adults (49), including eight ordinal items: general health (GH), physical functioning (PF), role physical (RP), bodily pain (BP), vitality (VT), social functioning (SF), mental health (MH), and emotional roles (RE). SF-8 has been proven to have good reliability and validity in a previous study (49). Responses were graded on a five-point Likert scale (1 = very poor, 5 = very good), indicating the minimum (5) and maximum (25) of possible SF-8 scores, and higher scores reflected a higher QoL. Sample items included “How would you describe your overall health?” A higher score reflected a lower quality of life among urban Chinese older adults The scale has an excellent internal consistency, with an overall Cronbach's alpha of 0.876 in this study.

### Data Analysis Methods

#### Preliminary Analyses

Statistical significance was determined to have been achieved for a two-tailed *p* < 0.05. All analyses were conducted using SPSS version 22.0 (IBM, BM SPSS Statistics for Windows). Pearson's correlation coefficients were calculated to examine the correlations among the variables: self-perceived stigma, attitude toward own aging, traditionality, and quality of life. Descriptive statistics of the demographic information and variables were indicated using the mean, standard deviation (SD), number (N), and percentage (%).

#### Moderator Analysis

In the present study, a series of associations among the variables were tested as well as to examine the moderating effects using Multiple Linear Regression Models (MLRM) and the predictive value of self-perceived stigma on the quality of life at different conditions. Two moderation analyses were performed by testing the significance of the interactions of self-perceived stigma and attitude toward own aging and the interactions of self-perceived stigma and traditionality. Statistical significance was also considered (50), then a simple slope analysis was conducted to visualize the interaction term.

## Results

### Demographic Information for Participants

Participants' demographic characteristics are shown in Table 1. The mean age of participants was 71.83 years (SD = 7.45, range = 60–96), and 54.19% were female. About 80.1, 9, and 18.7% were married, divorced, and had lost a spouse, respectively. In terms of education, 23.04% of participants had less than a full primary school education; 35.73% finished full primary school; 20.55% finished middle school; 12.96% finished high school; and 7.33% had higher education. In total, 44.37% of participants had a pension. About 76.70, 10.99, and 9.55% of participants defined their economic status as average, above average, and below average, respectively; 23.04% had an unsure status. Register refers to a person's origin or origins, and living conditions of their parents, which is divided into two group, namely a group of older adults migrated to current city and urban-local older adults.

**Table 1:** Demographic information of participants.

**Characteristics**	***N***	**%**
Sex		
Male	346	45.29
Female	414	54.19
Age		
≤ 65	167	21.86
66–70	217	28.40
71–75	161	21.07
76–80	115	15.05
≥81	104	13.61
Pension		
Yes	339	44.37
No	384	50.26
Unsure	41	5.37
Register		
Migrated to a city	437	57.20
Urban-local	326	42.67
Education categories		
No school	176	23.04
Primary school	273	35.73
Middle school	157	20.55
High school	99	12.96
Higher education	56	7.33
Unsure	3	0.39
Marital status		
Married	611	80.1
Divorce	9	1.2
Loss of spouse	143	18.7
Monthly income-cost	21	2.75
Below average	73	9.55
Average	586	76.70
Above average	84	10.99
Unsure	176	23.04

### Correlations Among Study Variables in the Total Sample (*N* = 764)

Pearson's correlation coefficients for continuous variables are shown in Table 2. Self-perceived stigma was positively correlated with attitude toward own aging (*r* = 0.405, *p* < 0.01) and traditionality (*r* = 0.118, *P* < 0.01) and negatively correlated with quality of life (*r* = −0.498, *p* < 0.01). Moreover, attitude toward own aging was negatively correlated with the traditionality (*r* = −0.073, *p* < 0.05) and quality of life (*r* = −0.418, *p* < 0.01).

**Table 2:** Means, standard deviation (SD), and correlations of continuous variables (*N* = 764).

**Variables**	**M**	**SD**	**1**	**2**	**3**	**4**
1 Self-perceived stigma	2.041	0.726	1			
2 Attitude toward own aging	2.955	0.559	0.405^**^	1		
3 Traditionality	3.201	0.755	0.118^**^	−0.073^*^	1	
4 Quality of life	4.205	0.580	−0.498^**^	−0.418^**^	−0.031	1

* *p < 0.05*;

** *p < 0.01*.

### Hierarchical Regression Analyses

**Figure 1:**
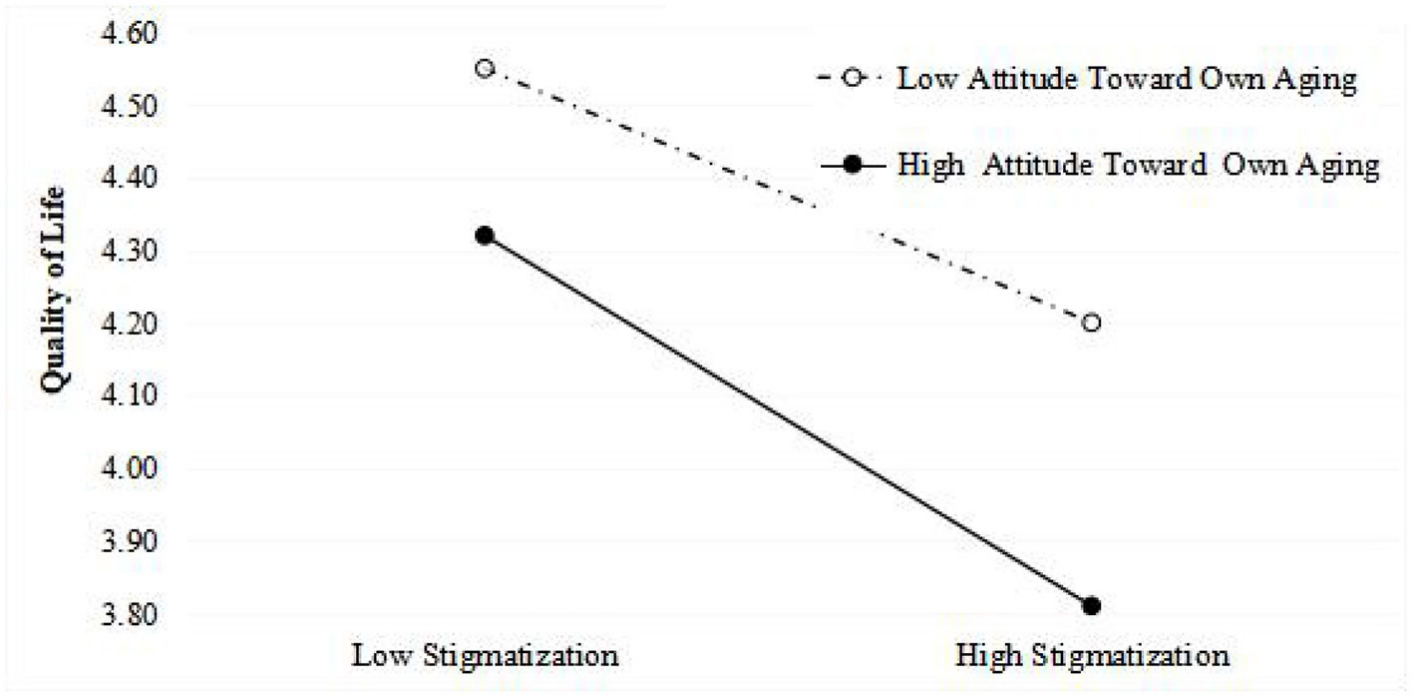
Simple slope diagram of the influence of the interaction between self-perceived stigma and attitude toward own aging on the quality of life.

**Figure 2:**
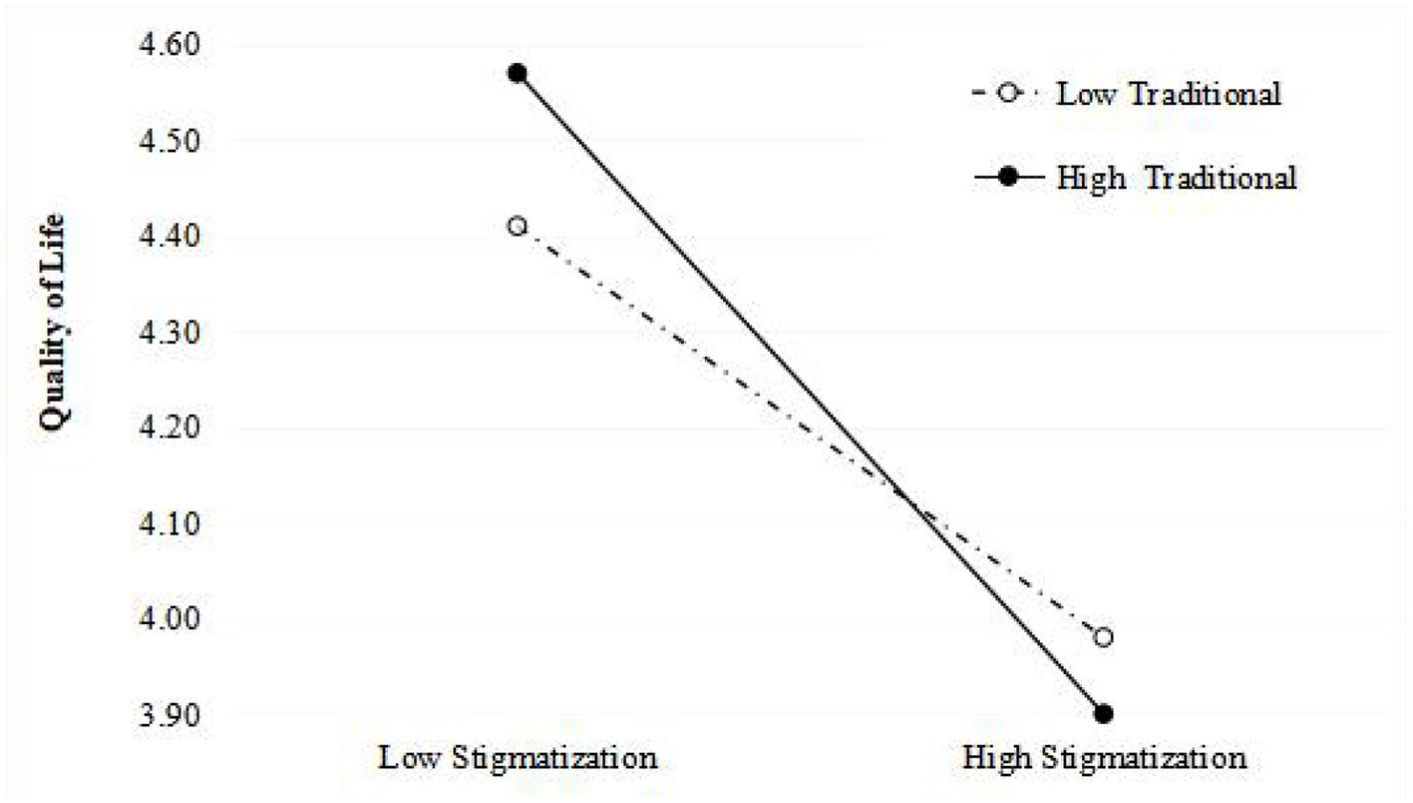
Simple slope diagram of the influence of the interaction between self-perceived stigma and traditionality on the quality of life.

Table 3 displays the results of a series of hierarchical regression analyses. The first step examined the influence of control variables, including sex, age, marital status, migrating status, retirement, and education level. In the second step, self-perceived stigma was found to be significantly and negatively related to the quality of life (β = –0.391, *p* < *0.01*). Similarly, attitude toward own aging was significantly and negatively associated with the quality of life (β = −0.211, *p* < *0.01)*. Self-perceived stigma and attitude toward own aging improved the model fits of quality of life (*R*^2^ = 0.334 and adjusted *R*^2^ = 0.237, *p* < *0.01*). The Self-perceived stigma × Attitude toward own aging interaction term was significantly and negatively associated with quality of life (β = −0.530, *p* < 0.01). Simple slope analysis revealed that when the attitude toward own aging was higher, the association between self-perceived stigma and quality of life became stronger. In other words, the strength of the impact of self-perceived stigma on quality of life was different in groups with negative (1 SD below the mean, *p* < 0.001) and positive (1 SD above the mean, *p* < *0.001*) attitudes toward own aging. For more legible and direct visualization of the results, the interaction is presented in Figure 1. Self-perceived stigma and traditionality improved the model fits of quality of life (*R*^2^ = 0.292 and *adjusted R*^2^ = 0.192, *p* < *0.01*). The Self-perceived stigma × Traditionality interaction term was also significantly and negatively associated with the quality of life (β = −*0.525, p* < *0.01*). Simple slope analysis revealed that when traditionality was higher, the association between self-perceived stigma and quality of life became stronger. In other words, the strength of the impact of self-perceived stigma on quality of life was different in groups with low (1 SD below the mean, *p* < *0.001*) and high (1 SD above the mean, *p* < *0.001*) traditionality. The interaction is visualized in Figure 2.

**Table 3:** Hierarchical linear regression models.

**Variables**	**Quality of life**				
	***M_**1**_(β)***	***M_**2**_(β)***	***M_**3**_(β)***	***M_**4**_(β)***	***M_**5**_(β)***
**Control variables**					
Sex	−0.083^*^	−0.095^**^	−0.087^**^	−0.087^**^	−0.082^**^
Age	−0.240^**^	−0.153^**^	−0.158^**^	−0.206^**^	−0.201^**^
Marital status	−0.068	−0.038	−0.037	−0.043	−0.047
Migrating status	0.085^*^	0.061	0.062	0.062	0.061
Retirement	−0.042	−0.012	−0.021	−0.056	−0.052
Education level	0.058	−0.027	−0.024	0.002	0.007
**Independent variable**					
Self-perceived stigma		−0.391^**^	0.025	−0.450^**^	−0.047
**Moderator variable 1**					
Attitude toward own aging		−0.211^**^	−0.013		
**Interaction 1**					
Self-perceived stigma × Attitude toward own aging			−0.530^**^		
**Moderator variable 2**					
Traditionality				−0.038	0.318^**^
**Interaction 2**					
Self-perceived stigma × Traditionality					−0.525^**^
*F*	14.155^**^	47.154^**^	43.218^**^	38.605^**^	35.914^**^
*R^2^*	0.097^**^	0.334^**^	0.341^**^	0.292^**^	0.301^**^
*ΔR^2^*	0.104^**^	0.237^**^	0.245^**^	0.192^**^	0.202^**^

*M1: control variables, including Sex, Age, Marital status, Migrating status, Retirement, and Education level*.

*M2: explains the influence of self-perceived stigma and attitude and toward own aging on Quality of Life*.

*M3: explains the influence of self-perceived stigma × attitude toward own aging on Quality of Life*.

*M4: explains the influence of self-perceived stigma and traditionality on Quality of Life*.

*M5: explains the influence of self-perceived stigma traditionality on Quality of Life*.

* *P < 0.05*;

** *P < 0.01 (two-tailed)*.

## Discussions

### Current Situation of Self-Perceived Stigma and Attitude Toward Own Aging Among Urban Chinese Older Adults

The results of this study showed that the self-perceived stigma (*M* = 2.041 ± 0.726) and attitude toward own aging (*M* = 2.955 ± 0.559) of urban Chinese older adults were lower than the median score, consistent with previous studies that suggested a less favorable perception of emotion experiences of older adults due to negative stereotypes about aging (51). Stigmatization related to age (i.e., ageism) is a widespread phenomenon in the modern industrial societies (26), as a self-concept threat, then which results in age-based stereotype threat about cognitive decline (52), further leading to a detrimental influence on older adults on their cognitive performance and self-perceived stigma, final generalizing negative attitude toward own aging (52).

Importantly, the current study indicates that self-perceived stigma and attitude toward own aging among urban Chinese older adults were very noticeable issues. It is common for urban Chinese older adults to endure with their own stigma and to keep a negative attitude toward their own aging, thus older adults are forced to embody socially acceptable labels to face the process of getting older. Theories such as stereotype embodiment (53) and social identity (54) theories have sought to provide a rationale for explaining the phenomena of self-perceived stigma, inferring that external stigma contributes to reinforcing internalized self-stereotypes. That is, aging self-stereotypes are more likely to increase negative expectations and attitudes toward older adults, which is prone to contribute to reduced daily activities frequency; this may result in decreased physical and emotional health (55). With the further assumption that a vicious cycle can develop as a potential possibility, wherein weakened cognitive and physical functioning of urban older adults likely leads to the formation of negative attitudes toward aging and self-perceived stigma (56). Further, social forces—driven by a series of media reports, mainstream culture, and marketing strategies—covertly shape public attitudes to aging by presenting implied negative narrative depictions toward urban older adults and the aging process. In turn, this further contributes to the formation of negative aging attitudes—such as uselessness, dependence, and hopelessness—and self-perceived stigma toward older individuals. As such, the current findings emphasize that targeted interventions alleviating urban older adults' self-perceived stigma and negative attitude toward own aging should be implemented to guard their quality of life.

### The Relationship Between Self-Perceived Stigma and Quality of Life

This study found that greater self-perceived stigma was related to lower quality of life among urban older adults in China, which is consistent with previous studies (57). Existing studies indicated that self-perceived stigma and prejudice were prone to lead to reduced cognitive and physical functioning (58), increased risk of depression and anxiety (59), and limited will to survive and quality of life among older adults (60).

### Attitudes Toward Own Aging Moderated the Relationship Between Self-Perceived Stigma and Quality of Life

This study found that negative attitudes toward own aging moderated the association between self-perceived stigma and quality of life. Results of the simple slope analysis showed that the stronger effect of self-perceived stigma on the quality of life among older adults exists along with more negative attitudes toward own aging, which refers to negative attitudes and beliefs toward older adults or toward the aging process itself (32). Older adults with a positive attitude toward their own aging are more likely to combat external stigma better, regulate their negative emotions openly, maintain health-promoting behaviors and lifestyles, and more easily make efforts to integrate into modern society (61). Older adults with negative attitudes toward their own aging tend to be more vulnerable facing stigma, leading to a further impairment in quality of life (62). Moreover, older adults with negative attitudes toward components of aging—such as disability or infirmity, which result in objective physical decline and social segregation—may also experience declines in their quality of life (63). In interpreting the interaction between self-perceived stigma and negative attitude toward own aging found in the present study, it can be speculated that older adults who recognize themselves as aging faster are more vulnerable to suffering negative concomitants of external stigma. In a “youth-centered” era, medical industries emphasize older age as a risk factor for health; this serves as both suggestion and autosuggestion for older adults regarding the process of aging. In turn, a cyclic establishment of self-perceived stigma is established, further destroying older adults' quality of life. Therefore, negative attitudes toward own aging are also potential threats that can render ones less immune to the harm of stigma. The findings from this study thus suggest that building positive attitudes among older adults toward aging may be a valid approach to minimize the degree of the negative impact of self-perceived stigma on the quality of life. Governments should initiate projects cultivating positive attitudes toward aging to better the quality of life of elderly people. Only when ageism is eliminated at all levels can a better age-friendly society exist.

### Traditionality Strengthened Moderated the Relationship Between Self-Perceived Stigma and Quality of Life

The present study highlighted that traditionality moderated the association between self-perceived stigma and quality of life, which confirmed the third hypothesis of this study. Traditionality seeps into all aspects of individual role definition in Chinese society (64) and contributes to affecting older adults' responses facing external stigma. Self-Determination Theory, which explains the results of this study (65), points out that people are willing to cultivate relationships with those who value their opinions and who are sensitive to their needs and wants (65), hinting that traditionalists are prone to experience decreased satisfaction of basic psychological needs rapidly (66) when they encounter negative external evaluation and stigmatization. This further results in reduced health-related and social-related quality of life (67). Influenced by Chinese cultural heritage, older adults with a high degree of traditionality often believe that younger individuals should show deference, be obedient, and be loyal to older groups (38). Often, older adults are used to maintaining their authority, thinking styles, and top-down communication, thus hindering fair and open interactions with younger individuals and leading to the increased probability of stigma from younger groups (35). Several organizational behavior studies confirmed that traditionality was conducive to organizational operation, personal satisfaction, and career success by increasing individual perceptions of procedural justice and job insecurity (68); further, this implies that traditionality is more likely to be suitable for a bureaucratic system that is characterized by a culture of hierarchy and compliance (41). However, traditionality does not always play a positive role for older adults in an aging society, especially in everyday life that emphasizes freedom and equality of communication. Conversely, in farming societies, getting older leads to a loss of power, authority, and autonomy. Older adults who are traditionalists are more likely to receive less psychological support and less likely to have positive relationships with others when facing stigma (38), resulting in decreased quality of life more than those with low degrees of traditionality. Moreover, older adults with modern characteristics, tendencies, or values are prone to deal with stigma with levity and openness; this contributes to one's flexible adjustment of their attitudes and behavioral responses, further buffering the self-inflicted damage of stigma on quality of life (64). Therefore, the current study posits that older adults who have traditionalist tendencies may hesitate to adjust their emotions and may enter a relatively negative psychological and social state, further strengthening the negative effect of self-perceived stigma on quality of life. Influenced by Confucian cultural tenets, traditionalism is deeply rooted in Chinese older adults and is not always conducive to positive outcomes, especially in modern eras (64). Therefore, this study's findings also suggest that an aging society must build a more open, inclusive, and diverse culture to alleviate the damage of stigma on the quality of life of older adults.

### Implications and Contributions

At last, implications for practitioners working with older adults are discussed as follow. This study contributes to some insights on the implications and contributions for formulating policies to deal with the Chins' aging tendency. Relatively low-cost, feasible strategies should serve as the basis of effective interventions to reduce ageism in the whole scope of society (1). Formal intervention programs regarding promoting and supporting self-management and self-care for elderly should be adopted to raise self-efficacy, then reducing their self-directed ageism (69). Moreover, modern lifestyle activities should be encouraged to foster the modern values of older adults (69). This study contributes to a new finding that individual cognition and cultural belief as the moderators determinate the strength of influence self-perceived stigma on the quality of life, which should facilitate establishing a public health and welfare policy for the most older adults, a group that has been increasing in China. Significantly, Chinese, Japanese, and Korean mostly rooted in Confucian values and ethics that are the central philosophic background for much of the culture in East Asia, particularly for understanding older adults' cognitive behavior (70).

A study in the Korean elderly revealed that there is the cultural characterization explanation of ageism and related coping processes among Korean elderly (71). However, a study conducting an international comparison found that the overall ageism score was lowest in Japan where favorable conditions for economic status, health status, and social participation are provided for older adults (72). From this, professional interventions and organizational interventions also be considered through teaching modern culture and beliefs in the community college to cultivate positive attitude toward aging for the elderly. Moreover, favorable conditions as a characterization of modernize city potentially protect older adults' self-efficacy for direct raising quality of life and indirect reducing their self-directed ageism.

Fortunately, the central government of China is already launching a comprehensive, in-depth reform to modernize the national governance system and capabilities to promote the quality of life and well-being of the elderly.

### Limitations

This study provided a new understanding regarding the association between self-perceived stigma and quality of life among urban Chinese older adults. However, the following limitations of this study must be emphasized. First, the data was collected through convenience sampling outdoors; this is prone to response bias, and unhealthy older adults, such as those with disabilities, may have been inadvertently omitted. Second, although much effort has been implemented to ensure a representative sample, it must be acknowledged that the effective response rate was not ideal. Third, a cross-sectional design cannot ascertain a causal relationship between self-perceived stigma and quality of life, suggesting that a longitudinal study is needed. Fourth, except for QOL (SF-8), other surveys had low Cronbach's alpha (<0.8) in the current study, indicating that more suitable or appropriate measuring instrument should be selected in future. Finally, considering that traditionality is a unique cultural phenomenon in China, further research should be conducted to test whether results from the current study are observable across different cultural contexts.

## Conclusion

In summary, the current study found that self-perceived stigma and attitude toward own aging of urban Chinese older adults were at a lower level. Moreover, both self-perceived stigma and attitude toward own aging were associated with a reduced quality of life, and both attitude toward own aging and traditionality can moderate the association between self-perceived stigma and quality of life. When negative attitudes toward own aging and traditionality were higher, self-perceived stigma had a greater effect on the quality of life. More importantly, older adults should be given more care and attention in daily life, and attitudes toward own aging and traditionality should be jointly intervened with to enhance the quality of life of older adults.

## Data Availability Statement

The original contributions presented in the study are included in the article/supplementary files, further inquiries can be directed to the corresponding author/s.

## Ethics Statement

The studies involving human participants were reviewed and approved by the Ethics Committee of Harbin Medical University (ECHMU). However, owing to the anonymous survey approach, written informed consent could not be obtained. Verbal informed consent to participates in this study was approved by the Institutional Review Board of Harbin Medical University and obtained from each of the participants on the front page of the questionnaire. Therefore, once a questionnaire was submitted successfully, we believed the consent of the nurse to participate in our study. Informed consent was obtained from all participants on the front page of self-administered questionnaires.

## Author Contributions

TS and S-EZ: conceptualization and software. TS, S-EZ, and X-hW: methodology. XC, K-yJ, and S-yC: formal analysis. S-EZ, M-yY, T-hL, and Y-qY: investigation. TS: resources and writing—original draft preparation. H-yY, C-xZ, and Y-pW: data curation. TS, S-EZ, X-hW, X-hH, and D-pC: writing—review and editing. X-hH and D-pC: visualization, supervision, and project administration. All authors have read and agreed to the published version of the manuscript.

## Conflict of Interest

The authors declare that the research was conducted in the absence of any commercial or financial relationships that could be construed as a potential conflict of interest.

## Publisher's Note

All claims expressed in this article are solely those of the authors and do not necessarily represent those of their affiliated organizations, or those of the publisher, the editors and the reviewers. Any product that may be evaluated in this article, or claim that may be made by its manufacturer, is not guaranteed or endorsed by the publisher.
